# Daily food insecurity is associated with diet quality, but not energy intake, in winter and during COVID-19, among low-income adults

**DOI:** 10.1186/s12937-022-00768-y

**Published:** 2022-03-24

**Authors:** Sara Jimenez Rincon, Nan Dou, Laura E. Murray-Kolb, Kristen Hudy, Diane C. Mitchell, Runze Li, Muzi Na

**Affiliations:** 1 Department of Nutritional Sciences, College of Health and Human Development, The Pennsylvania State University, University Park, PA USA; 2 Department of Biobehavioral Health, College of Health and Human Development, The Pennsylvania State University, University Park, PA USA; 3 Department of Nutrition Science, College of Health and Human Sciences, Purdue University, West Lafayette, IN USA; 4 Department of Statistics, Eberly College of Science, The Pennsylvania State University, University Park, PA USA

**Keywords:** Food insecurity, Low-income adults, Diet quality, Longitudinal, Ecological momentary assessment

## Abstract

**Background:**

Food insecurity (FI) is a dynamic phenomenon. Experiences of daily FI may impact dietary outcomes differently within a given month, across seasons, and before or during the COVID-19 pandemic.

**Objectives:**

The aims of this study were to investigate the association of short-term FI with dietary quality and energy 1) over six weeks in two seasonal months and 2) before and during the COVID-19 pandemic.

**Methods:**

Using an ecological momentary assessment framework on smartphones, this study tracked daily FI via the 6-item U.S. Adult Food Security Survey Module and dietary intake via food diaries in 29 low-income adults. A total of 324 person-days of data were collected during two three-week long waves in fall and winter months. Generalized Estimating Equation models were applied to estimate the daily FI-diet relationship, accounting for intrapersonal variation and covariates.

**Results:**

A one-unit increase in daily FI score was associated with a 7.10-point (95%CI:-11.04,-3.15) and 3.80-point (95%CI: -6.08,-1.53) decrease in the Healthy Eating Index-2015 (HEI-2015) score in winter and during COVID-19, respectively. In winter months, a greater daily FI score was associated with less consumption of total fruit (-0.17 cups, 95% CI: -0.32,-0.02), whole fruit (-0.18 cups, 95%CI: -0.30,-0.05), whole grains (-0.57 oz, 95%CI: -0.99,-0.16) and higher consumption of refined grains (1.05 oz, 95%CI: 0.52,1.59). During COVID-19, elevated daily FI scores were associated with less intake of whole grains (-0.49 oz, 95% CI: -0.88,-0.09), and higher intake of salt (0.34 g, 95%CI: 0.15,0.54). No association was observed in fall nor during the pre-COVID-19 months. No association was found between daily FI and energy intake in either season, pre-COVID 19, or during-COVID-19 months.

**Conclusion:**

Daily FI is associated with compromised dietary quality in low-income adults in winter months and during the COVID-19 period. Future research should delve into the underlying factors of these observed relationships.

**Supplementary Information:**

The online version contains supplementary material available at 10.1186/s12937-022-00768-y.

## Introduction

Food insecurity (FI) is defined as unreliable access to adequate food because of a lack of money or resources [1]. As of 2019, an estimated 10.5% of households in the United States were FI [2]. Due to the novel coronavirus 2019 (COVID-19) pandemic, however, rates of FI have almost tripled to 28.3% in U.S. households [3]. In low-income households, the prevalence of FI reached 44%, according to a national survey [4]. Low-income adults bore the brunt of the economic crisis; it forced individuals to maneuver flaws in the U.S. food system and existing economic disparities, thus likely increasing their risk to have FI [5].

A poor diet has been associated with chronic FI and has been thought to contribute to the pathology of numerous noncommunicable diseases such as diabetes, coronary heart disease, and obesity [6–9]. An aspect of FI that is not well represented in the literature, however, is the cyclical nature of unreliable food access and its relation to diet quality. Food security is a dynamic rather than a static phenomenon within a given month [10] and across seasons [11]. Other short-term shocks, such as changes in employment and the COVID-19 pandemic, have been found to influence the likelihood of FI [4, 12]. In addition, many studies investigating the association of short-term FI with dietary outcomes have been carried out [13], where seasonal, short-term shocks in food supply, access, and utilization are common [14, 15]. Though the literature has noted temporal shifts in food security [11, 16], day-to-day FI experiences and their potential consequences on dietary intake within a given month, as well as comparisons across different time periods of the year of potentially different risk of FI (e.g. by season, before and during COVID-19 pandemic), have been generally understudied.

This study aimed to fill the following research gaps 1) to investigate how daily FI is associated with daily dietary quality and daily calorie intake in low-income adults in Central Pennsylvania; and 2) to investigate the relationships between daily FI and daily dietary intake by season and by pandemic status (i.e. before and during the COVID-19 pandemic).

## Methodology

### Subjects and study design

Data for this study were collected as part of the Food ‘N Mood Study, a pilot study carried out in Central Pennsylvania from September 2019 to March 2021 that aimed to examine the impact of daily FI on diet, mood and heart rate variability. Participants were recruited from selected locations serving low-income populations, such as the office for the Special Supplemental Program for Women, Infants and Children (WIC), the county assistance office, food banks, and Head-Start childcare services, in rural areas within Centre, Clearfield, Clinton, Blair, Elk, Bradford-Tioga, Huntingdon, and Snyder-Union-Mifflin counties. Healthy adults aged 20 to 50 years with a household income that fell below 185% of the Federal Poverty Line (FPL) were eligible to be enrolled. Exclusion criteria included adults who were non-English speakers, who had physical, mental or emotional disabilities, who used medications or experienced medical conditions known to affect heart rate variability, or who had disabled people as part of their household. Females who were pregnant or who had reached menopause were also excluded from the study.

An ecological momentary assessment (EMA) model on smartphones was used to collect daily FI and dietary data. Participants completed their data collection over two 3-week-long waves (from the 2nd to the 4th week of the month), covering one month in the fall season (September, October, or November) in 2019 or 2020, and another month in the winter season (February or March) in 2020 or 2021. On their devices, participants filled out a daily evening survey that asked about FI experiences in the past twenty-four hours. On Sunday, Monday, and Tuesday of the study weeks, participants were asked to report dietary intake on a food record module. The study protocol was approved by The Pennsylvania State University Institutional Review Board, University Park, Pennsylvania and the National Center for Advancing Translational Sciences (NCATS).

### Assessment of daily FI

Daily FI status was measured using an adapted 6-item U.S. Adult Food Security Survey Module [17]. Each day, participants were asked about the food situations that they encountered in the past twenty-four hours. The food situations included: due to a lack of money, the participant ‘was worried about food running out,’ ‘did not eat a balanced meal,’ ‘cut meal size or skipped a meal,’ ‘ate less,’ ‘was hungry but did not eat,’ and ‘did not eat for a whole day.’ A daily FI score was calculated as the sum of the six survey answers, yielding a total score ranging from 0 (experiencing no food insecure situations) to 6 (experiencing all food insecure situations).

### Assessment of dietary intake

Food records were collected on three days (Sunday, Monday, and Tuesday) per week for three weeks in both fall and winter seasons using the provided smartphones. Built-in notifications at the end of each survey module were used to remind participants to complete their food diary. Participants were encouraged to record their food intake at the moment when they were eating or right after they ate. The application queried information on food items, food amount, food preparation, and timing and location of the food intake. Participants recorded dietary intake using free-text entries, and a trained research dietitian from the Diet Assessment Center at Penn State downloaded the dietary intake data from the research portal and re-entered into Nutrition Data System for Research software (NDSR, versions 2019 and 2020, the Nutrition Coordinating Center (NCC), University of Minnesota, Minneapolis, MN). To improve the accuracy of the dietary data, this trained research dietitian reviewed the NDSR-entered food record with the participant over the phone to confirm portion sizes and foods consumed on the day following the date of dietary data entry (i.e. a follow-up phone call was made on Monday to verify dietary intake information entered on Sunday). This interview verification process is known as ‘record-assisted recall’ and has been validated in a previous study [18]. Dietary intake data were analyzed using NDSR. The NDSR time-related database updates analytic data while maintaining nutrient profiles true to the version used for data collection.

Diet quality was assessed using the Healthy Eating Index 2015 (HEI-2015) using the simple HEI scoring algorithm method [19–22]. The index contains 13 components, including total fruits, whole fruits, total vegetables, greens and beans, total protein foods, seafood and plant proteins, whole grains, dairy, fatty acids, refined grains, sodium, added sugars, and saturated fats. Scores are calculated proportionately according to the intakes between the minimum and maximum standards, aligning with the 2015–2020 Dietary Guidelines for Americans. The maximum possible HEI-2015 score is 100 [19–21].

### Assessment of demographic and socioeconomic characteristics

At baseline, a background survey was administered to collect demographic, socioeconomic, and health characteristics, including age, gender, race/ethnicity, height, weight, education, employment status, marital status, household size, number of children under 18 years in the household, total annual household income, enrollment in food assistance programs, and chronic household FI in the past 12 months. Body mass index (BMI) was calculated based on self-reported weight and height by weight (kg) / height^2^ (m). Chronic FI status in the past 12 months was measured using the 10-item U.S. Adult Food Security Survey Module [23]. According to the Department of Health and Human Services’ definition, poverty status was categorized as < 130% FPL or >  = 130% and < 185% FPL considering the total household income and household size [24, 25].

### Statistical analysis

All statistical analyses were performed in R (Version 4.0.5; R Foundation for Statistical Computing, Vienna, Austria). To summarize the characteristics of participants, means and standard deviations were reported for age, household size, the number of children under 18 in the household, BMI, HEI scores, and total energy. Proportions were reported for gender, race/ethnicity, education, employment, marital status, poverty status, and enrollment in food assistance programs. The differences in HEI scores and total energy intake were tested using the Wilcoxon Mann–Whitney nonparametric test because of their skewed distribution. Instead of analyzing FI status and dietary intake for each participant, we used person-days of information to examine the day-to-day associations between FI and dietary intake. Specifically, our study included completed person-days of information on daily FI and dietary intake three days per week (Sunday, Monday, and Tuesday) in the study months. The Generalized estimating equation (GEE) models were used to explore the associations between FI and total calorie intake, diet quality, and food groups intake in 1) fall and winter seasons, and 2) in pre-COVID-19 (September, October, and November in 2019, and February in 2020) and during-COVID-19 months (March in 2020, October and November in 2020, and February in 2021). Covariates, including gender [26, 27], race/ethnicity [28, 29], employment [30, 31], poverty status [32, 33], weekdays [34, 35], study weeks [10, 36], seasons [11, 37], and COVID-19 months [4, 38] were adjusted in the analysis, given their known associations with both FI and dietary intake. Seasons were adjusted in models examining the FI-diet associations by COVID-19 months, and COVID-19 months were adjusted in models by seasons. Total calorie intake was further adjusted in these models when estimating the associations between FI and the intake of individual food groups. The autoregressive correlation structure “AR1” was applied with GEE models accounting for within-person correlation in dietary intake [39]. Because our study included 86.2% female participants, we also conducted sensitivity analysis to compare the FI-diet associations between all participants and female participants.

## Results

Overall, 28 participants were recruited in fall 2019/2020 and 25 participants were followed up in winter 2020/2021. One participant was recruited in winter 2020/2021. Sample characteristics are presented in Table 1. The mean age of participants was 36.3 years. The majority (86.2%) of participants were female, had a college degree or above (82.8%), were employed (72.4%), and over half of participants were White (69.0%). About half of the sample (48.3%) was married.

**Table 1:** Characteristics of the low-income participants (< 185% of FPL) enrolled in the Food ‘N Mood Study^a,b^

	**All participants (*n* = 29)**
**Age**	36.3 (7.1)
**Female**	25 (86.2%)
**Race / ethnicity**	
White or Caucasian	20 (69.0%)
Black or African American, Asian, and Other	9 (31.0%)
**Education**	
Less than high school, high school/GED, OR some college	5 (17.2%)
College and above	24 (82.8%)
**Employment**	
Employed	21 (72.4%)
Unemployed	8 (27.6%)
**Marital Status**	
Married, with partner	14 (48.3%)
Single, divorced, widowed	15 (51.7%)
**Household Size**	4.0 (1.7)
**Number of children under 18 years in the household**	2.2 (1.7)
**Poverty status**	
$\hspace{1em}\ge$ 130% FPL and < 185% FPL	13 (44.8%)
$\hspace{1em}<$ 130% FPL	16 (55.2%)
**Food assistance programs, SNAP / Food Stamps**	8 (27.6%)
**BMI, kg/m**^**2**^** (*N* = 22)**	27.8 (8.4)
**Chronic FI**	13 (44.8%)

^a^*Abbreviation*: *BMI* body mass index, *FPL* federal poverty line, *SNAP* Supplemental Nutrition Assistance Program, *HEI*, healthy eating index

^b^Mean (SD) were reported for continuous variables including age, household size, number of children under 18 years in the household, and BMI. N (%) were reported for categorical variables including gender, race/ethnicity, education, employment, marital status, poverty status, and participation in food assistance programs

Across fall and winter, a total of 330 person-days (response rate 67.9%), or an average of 11.3 days per participant of information on daily FI and dietary records were collected. One hundred and eighty-one (response rate 69.3%) or an average of 6.5 days per participant of dietary records were collected in the fall, and 149 (response rate 63.6%) or an average of 5.7 days per participant of dietary records were collected in the winter. After excluding dietary records with daily calorie intake less than 500 kcal or greater than 4000 kcal (n of person-days = 6) as suggested by Willett et al. [40], a total of 324 person-days of dietary information were included in our analysis. The distribution of HEI scores and total energy intake are presented in Table 2. Out of the 100 possible points, the mean (SD) HEI score over study days (ranges from 1–9) was 44.8 (13.7) in the fall months and 45.9 (13.3) in the winter months. The mean (SD) HEI score was 46.6 (14.7) and 42.8 (10.6) before and during COVID-19, respectively. Mean (SD) energy was 1663.1 kcal (676.5) and 1532.4 kcal (585.2) in the fall and winter months, respectively. The mean (SD) energy was 1637.3 kcal (618.0) before COVID-19 and 1539.8 kcal (677.3) during COVID-19. Higher total energy intake and HEI scores were observed before COVID-19 as compared to those during COVID-19. There was no significant differences in total energy or HEI scores between the fall and the winter month.

**Table 2:** Distribution (mean (SD)) of HEI-2015 scores and total energy across fall and winter seasons, and pre- and during COVID-19 pandemic^a^

	**All**	**By season**			**By COVID period**		
		**Fall**	**Winter**	***P*-value**	**Before Covid-19**	**During Covid-19**	***P*-value**
**N of person-days**	324	178	146		214	110	
**Total energy, kcal**	1604.2 (639.4)	1663.1 (676.5)	1532.4 (585.2)	0.09	1637.3 (618.0)	1539.8 (677.3)	**0.05**
**HEI, score**	45.3 (13.5)	44.8 (13.7)	45.9 (13.3)	0.35	46.6 (14.7)	42.8 (10.6)	**0.01**

^a^Between-group differences in HEI score and total energy intake were tested by Wilcoxon Mann–Whitney nonparametric test because they were not normally distributed

Table 3 summarizes the association between daily FI and dietary outcomes. In the overall sample, we found daily FI was significantly associated with decreased HEI-2015 score (beta-coefficient = -2.40, 95% CI: (-4.80, -0.01), *P* = 0.05), intake of total fruit (beta-coefficient = -0.10 cups, 95% CI: (-0.19, -0.01), *P* = 0.02), greens and beans (beta-coefficient = -0.05 cups, 95% CI: (-0.11, -0.01), *P* = 0.05), and whole grains (beta-coefficient = -0.30 oz, 95% CI: (-0.51, -0.09), *P* < 0.004), as well as increased intake of dairy (beta-coefficient = 0.31 cups, 95% CI: (0.11, 0.51), *P* < 0.002) and refined grains (beta-coefficient = 0.44 cups, 95% CI: (0.14, 0.74), *P* < 0.004). There was no association between daily FI and energy intake. Across seasons, a one unit increase in the daily FI score was associated with a decrease in the HEI-2015 score by 7.10 points (95% CI: -11.04, -3.15) in the winter (*P* < 0.001). Specifically, whole fruit intake decreased by 0.17 cups (95% CI: (-0.32, -0.02), *P* = 0.03), total fruit decreased by 0.18 cups (95% CI: (-0.30, -0.05), *P* < 0.007), and whole grain intake decreased by 0.57 oz (95% CI: (-0.99, -0.16), *P* < 0.007). Daily FI was also associated with increased intake of refined grains, as every one unit increase in the daily FI score was associated with an increase in 1.05 oz (95% CI: (0.52, 1.59), *P* < 0.001) higher intake of refined grains. In the fall, however, daily FI was not associated with overall diet quality, quantified through HEI-2015 score, although a slight decrease in greens and beans intake (beta-coefficient = -0.05 cups, 95% CI: (-0.10, -0.01), *P* = 0.05), total protein (beta-coefficient = -0.61 oz, 95% CI: (-1.09, -0.14), *P* = 0.01) and increase in dairy intake (beta-coefficient = 0.44 cups, 95% CI: (0.14, 0.73), *P* < 0.004) were observed. Daily FI was not significantly associated with changes in energy intake in either the fall or the winter seasons.

**Table 3:** The adjusted associations between daily food insecurity score and energy intake, diet quality, and HEI component scores in the overall sample and in sample stratified by season and by COVID-19 period^a^

	**All**^**b**^		**By season**				**By COVID period**			
			**Fall**^**c**^		**Winter**^**c**^		**Before Covid-19**^**d**^		**During Covid-19**^**d**^	
	b (95% CI)	*P*-value	b (95% CI)	*P*-value	b (95% CI)	*P*-value	b (95% CI)	*P*-value	b (95% CI)	*P*-value
N of person-days	324		178		146		214		110	
**Total diet**										
Total energy, kcal^e^	-7.58 (-125.50, 110.30)	0.90	2.35 (-144.00, 148.90)	0.98	-39.60 (-216.80, 137.70)	0.66	69.86 (-67.30, 207.00)	0.32	-136.90 (-296.00, 22.10)	0.09
HEI, score^e^	**-2.40** **(-4.80, -0.01)**	**0.05**	-1.05 (-3.87, 1.79)	0.47	**-7.10** **(-11.04, -3.15)**	** < 0.001**	-2.45 (-6.21, 1.31)	0.20	**-3.80** **(-6.08, -1.53)**	** 0.001**
**Food and food group**										
Total fruit, cup	**-0.10** **(-0.19, -0.01)**	**0.02**	-0.07 (-0.17, 0.03)	0.17	**-0.17** **(-0.32, -0.02)**	**0.03**	**-0.20** **(-0.32, -0.07)**	** 0.003**	0.08 (-0.07, 0.22)	0.30
Whole fruit, cup	-0.05 (-0.14, 0.03)	0.23	-0.06 (-0.16, 0.04)	0.28	**-0.18** **(-0.30, -0.05)**	** 0.007**	**-0.16 (-0.28, -0.04)**	** 0.009**	0.09 (-0.08, 0.25)	0.31
Total vegetable, cup	0.04 (-0.21, 0.29)	0.78	0.08 (-0.22, 0.37)	0.62	-0.11 (-0.39, 0.17)	0.44	-0.13 (-0.39, 0.13)	0.34	0.23 (-0.03, 0.49)	0.08
Greens and beans, cup	**-0.05** **(-0.11, -0.01)**	**0.05**	**-0.05** **(-0.10, -0.01)**	**0.05**	-0.07 (-0.15, 0.01)	0.09	**-0.07** **(-0.13, -0.02)**	**0.01**	-0.07 (-0.14, 0.01)	0.10
Whole grains, oz	**-0.30** **(-0.51, -0.09)**	** 0.004**	-0.16 (-0.37, 0.06)	0.16	**-0.57** **(-0.99, -0.16)**	** 0.007**	**-0.29** **(-0.57, -0.01)**	**0.04**	**-0.49** **(-0.88, -0.09)**	**0.02**
Dairy, cup	**0.31** **(0.11, 0.51)**	** 0.002**	**0.44** **(0.14, 0.73)**	** 0.004**	0.09 (-0.32, 0.50)	0.67	**0.31** **(0.04, 0.58)**	**0.02**	0.18 (-0.03, 0.39)	0.10
Total protein, oz	-0.44 (-1.02, 0.15)	0.14	**-0.61** **(-1.09, -0.14)**	**0.01**	-0.20 (-1.10, 0.70)	0.67	-0.41 (-1.10, 0.29)	0.25	-0.40 (-1.00, 0.20)	0.20
Seafood/plant protein, oz	0.03 (-0.37, 0.42)	0.90	0.08 (-0.37, 0.53)	0.74	-0.03 (-0.41, 0.34)	0.86	0.24 (-0.33, 0.82)	0.41	-0.41 (-0.95, 0.12)	0.13
Refined grains, oz	**0.44** **(0.14, 0.74)**	** 0.004**	0.17 (-0.19, 0.54)	0.36	**1.05** **(0.52, 1.59)**	** <0.001**	0.35 (-0.07, 0.76)	0.10	0.60 (-0.03, 1.24)	0.06
Salt, g	0.12 (-0.08, 0.31)	0.24	0.07 (-0.17, 0.30)	0.57	0.17 (-0.07, 0.41)	0.16	-0.04 (-0.23, 0.14)	0.63	**0.34** **(0.15, 0.54)**	** < 0.001**
Added sugars, % of energy	-14.13 (-31.69, 3.43)	0.11	-14.30 (-51.68, 23.08)	0.45	1.35 (-55.57, 58.28)	0.96	-17.89 (-41.05, 5.29)	0.13	-5.36 (-38.05, 27.33)	0.75

^a^*CI *confidence interval, *HEI *healthy eating index

^b^Adjusted for gender, race, employment, poverty status, COVID-19 months, seasons, total energy intake, study weeks, and weekdays

^c^Adjusted for gender, race, employment, poverty status, COVID-19 months, total energy intake, study weeks, and weekdays

^d^Adjusted for gender, race, employment, poverty status, seasons, total energy intake, study weeks, and weekdays

^e^GEE models examining the association of daily FI scores and total energy intake and HEI score were not adjusted for total energy intake

When stratifying data by COVID-19 months, we found that daily FI was not associated with energy intake before or during COVID-19. FI was associated with decreased HEI-2015 score during (beta-coefficient = -3.80, 95% CI: (-6.08, -1.53), *P* < 0.001), but not before the COVID-19 pandemic (beta-coefficient = -2.45, 95% CI: (-6.21, 1.31), *P* = 0.20). During COVID, daily FI was associated with lower intake of whole grains by 0.49 oz (95% CI: (-0.88, -0.09), *P* = 0.02), and higher intake of salt by 0.34 g (95% CI: 0.15, 0.54), *P* < 0.001). Before COVID, there were a few individual food groups that were found associated with daily FI, including decreased intake of total fruit (beta-coefficient = -0.20 cups, 95% CI: (-0.32, -0.07), *P* < 0.003), whole fruit (beta-coefficient = -0.16 cups, 95% CI: (-0.28, -0.04), *P* < 0.009), greens and bean (beta-coefficient = -0.07 cups, 95% CI: (-0.13, -0.02), *P* = 0.01), and whole grains (beta-coefficient = -0.29 oz, 95% CI: (-0.57, -0.01), *P* = 0.04), and increased intake of dairy (beta-coefficient = 0.31 cups, 95% CI: (0.04, 0.58), *P* = 0.02).

By excluding person-days of information on FI and dietary intake from male participants, the sensitivity analysis showed the robust associations between daily FI and HEI-2015 scores in winter and during COVID-19 compared to the main findings (Supplemental Table 1). Specifically, a one-unit increase in daily FI score was associated with 6.18 (95% CI: (-10.26, -2.10), *P* < 0.003) and 3.87 (95% CI: (-6.19, -1.55), *P* < 0.001) points decrease in HEI-2015 scores in winter and during COVID-19, respectively. The findings regarding the daily FI—food group associations were largely consistent as well. The only exception was in the female-only sample, where we additionally observed a significant increase in dairy consumption related to daily FI during the COVID-19 period (beta-coefficient: 0.24 cups, 95% CI: (0.08, 0.41), *P* < 0.003). This association was insignificant in the original analysis (beta-coefficient = 0.18 cups, 95% CI: (-0.03, 0.39), *P* = 0.10).

## Discussion

This study sought to explore the relationship between daily FI and day-to-day diet in low-income adults. We analyzed the longitudinal, intrapersonal FI and diet data collected through an EMA framework using smartphones. Though overall mean HEI was slightly higher in the winter than fall, we found a lower overall diet quality score associated with the number of daily FI experiences in the winter and during the COVID-19 months. Changes in the consumption of a few food groups seem to drive the compromised dietary quality during these vulnerable periods of the year, including the decreased intake of whole fruit, total fruit, and whole grains in the winter season, and decreased intake of whole grains and total proteins and increased intake of salt during the COVID-19 months. We did not see a statistically significant association between daily FI and energy intake in the overall sample or in stratified analyses. Our empirical data from this study showed that daily FI experienced during particular times of the year, such as in the winter and in pandemic months, is associated with poorer day-to-day dietary quality, but not energy intake, in low-income adults.

After adjusting for total energy intake and other socio-demographic covariates, our results display a worsened dietary pattern in winter than fall season, and during COVID-19 than pre-pandemic. The mean HEI-2015 scores in our sample were 44.8 (SD) in the fall and 45.9 (SD) in the winter, which were even lower than the 57-point average for Americans living under 131% FPL and the 57-point average for Americans living between 131 and 350% FPL [41]. The recommended daily intake for fruit and whole grains for adult women is 1.5 to 2 cups and 5 to 7 oz respectively [42, 43]. For every additional FI experience in the winter, participants’ daily fruit intake fell 9.0–12.0 percentage points (0.18 cups) from the ideal dietary pattern, and daily whole grains intake fell 8.1–11.4 percentage points (0.57 oz) from the ideal dietary pattern. For every one unit increase in FI in COVID-19 months, participants’ daily intake of whole grains fell 7.0–9.8 percentage points (0.49 oz) from the ideal dietary pattern, respectively. Mild to severe cases of FI thus may compromise the nutrient density of an individual’s diet and contribute to diet-related diseases and deficiencies.

The low HEI-2015 scores observed in our sample reflect poor dietary quality. We found that participants who experienced greater daily FI had the lowest diet quality, probably due to decreased available income for food [44]. Additionally, areas where FI rates are high tend to have less access to nutrient-dense foods while simultaneously having readily accessible processed, low-cost foods [45, 46]. Single day energy intakes were well below the national average for individuals of the same age, sex, and income, and these differences in energy could have accounted for some of the differences in HEI-2015 in this study compared to national data. Rating lower on food procurement, preparation skills, and self-efficacy related to healthy eating can partially explain lower diet quality among individuals with FI, though this mostly applies to populations with lower education levels than those of the current sample [47, 48]. FI is thus related to lower diet quality at multiple levels and is a complex issue to tackle.

One of the main findings revealed in this study was the differential FI-diet quality association conditioned on the time of the year. Seasonal differences in FI risk and dietary outcomes have been discussed in prior research. Among low-income individuals, changes in heating and cooling costs can force economic tradeoffs between food expenses and other basic necessities, what was documented as a “heat or eat” effect by Bhattacharya et al. [11, 49]. As such, low-income families may turn to low-nutrient density diets that have a lower cost and higher energy per serving than nutrient-dense foods, such as fruit and whole grains [50]. Additionally, coping strategies for FI include drawing on alternate food sources, such as food pantries, federal food assistance, and social support, but these may be inhibited by winter weather conditions [51]. Some market research indicates that weather may affect shoppers’ frequency and basket size [52, 53]. People’s positive feelings on good weather may motivate their shopping behaviors. Severe weather event, such as rain and snow decrease both the shopping trip and basket size. Low-income individuals living in central Pennsylvania, for example, may thus experience lower access to a diverse, healthful diet due to the above-mentioned winter-related barriers.

We found that daily FI was related to poorer overall diet quality during the months of, but not before, the COVID-19 pandemic. Findings in the literature may underlie this observed relationship. Due to mass quarantine, the food system was disrupted at several levels which impacted food availability and food prices in many communities [54, 55]. Not only was food production and transportation impacted, but “panic buying” could have limited food supply for consumers [54]. Loss of employment due to the pandemic could have reduced the affordability of healthy food for many people [56]. Mass quarantine also reduced social contact to a massive extent, which may have led to limited access to alternate food sources and social support, and fear of COVID-19 infection could have restricted individuals’ willingness to obtain food [56]. The COVID-19 pandemic may have impacted the participants of this study in a similar manner to what has been observed in the literature. As to why the associations were only observed during, but not before, the pandemic, we compared the food insecurity experience (pre-pandemic vs pandemic) and found more participants worried about running out of food before the pandemic (3.3% vs 0%); however, during the pandemic, more people reported eating unbalanced meals (36.9% vs 46.4%), skipping meals (2.3% vs 7.3%), eating less (0.9% vs 8.2%), and suffering from hunger (0.5% vs 3.6%). It seems that more participants suffered from decreased food quality and reduced food quantity during the COVID period, which may explain why significant associations were only seen in the pandemic subsample.

Though diet quality differed across different times of the year in this sample of low-income individuals, food energy did not fluctuate. It is possible that underreporting of food intake by participants or, in some cases, missing or incomplete data could have impacted the study results. Nevertheless, previous research has found no differences in calorie intake among individuals with chronic FI and food secure individuals, ostensibly attributed to a higher per-meal energy, increased snack energy, frequent consumption of energy-dense foods, and a higher fat intake among individuals with FI [57–59]. Due to economic constraints, individuals with FI may turn to purchasing the cheapest calories possible, thus meeting or approaching energy requirements, though not overconsuming food since it is a scarce resource (36). Our study suggested that FI experienced on a daily basis by low-income adults mostly reflected experiences of mild to moderate, rather than severe, FI which is associated with reduced quality, but not energy content of diet.

There are some limitations of the study to note. While our study found the differences in the association of daily FI and diet quality by seasons and over the pandemic, the pilot data may not allow us to further explain the differences. Future large cohort studies with daily FI and dietary intake data are needed to further explore the differences observed in this pilot study. This study cannot establish a causal relationship between FI and diet quality. Participants may have underreported their dietary intake in our study, that has been observed in previous studies among adults [60]. However, the underreported dietary intake may not have any impacts on the study findings given the commonality of the issue as well as the follow-up calls by a trained dietitian providing a more complete dietary intake. There remains a question about the representativeness of the study sample given that it is majority female, White, employed, and college-educated. Individuals with FI who have higher levels of education may score higher on dimensions of food literacy (knowledge of nutrition, budgeting, and food selection) so the effects of FI may differ among differently educated populations [47]. When we excluded male participants, our sensitivity analysis found consistent FI-total-energy and FI-HEI associations (Supplemental Table 1). Future studies are warranted to verify if findings can be replicated in males. Additionally, the response rate of 67.9% for both FI and dietary measures is moderate, and therefore may not entirely capture the variables and relationships of interest. This is a pilot study with a small, highly educated sample, and replication in larger representative samples is necessary for generalization.

Several strengths of this study are also to be recognized. For example, the intensive longitudinal data being collected over 3 weeks in 2 seasons that allowed for investigating the dynamics of FI-diet relationships over time. Additional strengths include the use of smartphones for timely gathering of daily FI and dietary data, and the collection of missing dietary details not gathered from the smartphone application through a follow-up phone call by a trained dietitian. This pilot study achieved the goal of tracking daily FI and diet in the real-world living environment of a sample of low-income adults.

## Conclusion and future directions

This longitudinal pilot study utilized a novel EMA framework on smartphones to collect daily FI and diet data across two different seasons and before and during the COVID-19 pandemic. We identified two periods of time, in the winter and during the initial months of the COVID-19 pandemic, as the most vulnerable time periods where dietary quality, but not caloric intake, was significantly reduced in relation to daily FI. This study provides some preliminary evidence of external validity when we associate daily FI measures with diet quality and potentially identifies critical points of intervention to improve the overall well-being of individuals with FI. Future research is expected to verify the study findings, investigate the factors underlying observed differences in the FI-diet quality relationship by season and by COVID-19 pandemic status, as well as expand on the observed relationship between daily FI and energy intake in larger etiological studies.

## Supplementary Information

**Additional file 1: Table S1.** The adjusted associations between food insecurity score and food/nutrient intake, diet quality, and HEI component scores in females.

## Data Availability

The datasets generated during and/or analyzed during the current study are not publicly available due to their containing information that could compromise the privacy of research participants but are available from the corresponding author on reasonable request.

## Abbreviations

FI: Food Insecurity
FPL: Federal Poverty Line
EMA: Ecological Momentary Assessment
NDSR: Nutrition Data System for Research
HEI-2015: Healthy Eating Index-2015
BMI: Body Mass Index
GEE: Generalized Estimating Equation

## References

[1] Ziliak CGJP (2015). Food Insecurity And Health Outcomes. Health Aff.

[2] Coleman-Jensen A, Rabbitt MP, Gregory CA, Singh A. Household food security in the United States in 2019, Economic Research Report 275. Washington (DC): USDA Economic Research Service; 2020; [cited 8 August, 2021]. Available from: https://www.ers.usda.gov/webdocs/publications/99282/err-275.pdf?v=310.7.

[3] United States Census Bureau: Recent Food Insufficiency for Households, by Additional Food Related Household Characteristics: United States. In Week 28 Household Pulse Survey: April 14 – April 26, 2021. [cited 8 August, 2021]. Available from: https://www.census.gov/data/tables/2021/demo/hhp/hhp28.html.

[4] Wolfson JA, Leung CW (2020). Food Insecurity During COVID-19: An Acute Crisis With Long-Term Health Implications. Am J Public Health.

[5] Leddy AM, Weiser SD, Palar K, Seligman H (2020). A conceptual model for understanding the rapid COVID-19-related increase in food insecurity and its impact on health and healthcare. Am J Clin Nutr.

[6] Larson N, Laska MN, Neumark-Sztainer D (2020). Food Insecurity, Diet Quality, Home Food Availability, and Health Risk Behaviors Among Emerging Adults: Findings From the EAT 2010–2018 Study. Am J Public Health.

[7] Robaina KA, Martin KS (2013). Food insecurity, poor diet quality, and obesity among food pantry participants in Hartford CT. J Nutr Educ Behav.

[8] Seligman HK, Bindman AB, Vittinghoff E, Kanaya AM, Kushel MB (2007). Food insecurity is associated with diabetes mellitus: results from the National Health Examination and Nutrition Examination Survey (NHANES) 1999–2002. J Gen Intern Med.

[9] Smith MD, Coleman-Jensen A (2020). Food insecurity, acculturation and diagnosis of CHD and related health outcomes among immigrant adults in the USA. Public Health Nutr.

[10] Gassman-Pines A, Schenck-Fontaine A (2019). Daily Food Insufficiency and Worry among Economically Disadvantaged Families with Young Children. J Marriage Fam.

[11] Nord M, Kantor LS (2006). Seasonal variation in food insecurity is associated with heating and cooling costs among low-income elderly Americans. J Nutr.

[12] Webb P, Coates J, Frongillo EA, Rogers BL, Swindale A, Bilinsky P. Measuring household food insecurity: why it’s so important and yet so difficult to do. J Nutr. 2006;136:1404s–8s. 10.1093/jn/136.5.1404S 16614437

[13] Jackson AM, Weaver RH, Iniguez A, Lanigan J (2022). A lifespan perspective of structural and perceived social relationships, food insecurity, and dietary behaviors during the COVID-19 pandemic. Appetite.

[14] FAO, Ifad, UNICEF, WFP, WHO: The State of Food Security and Nutrition in the World (2021). Transforming food systems for food security, improved nutrition and affordable healthy diets for all.

[15] Fao I, UNICEF, WFP and WHO.: The State of Food Security and Nutrition in the World (2021). Transforming food systems for food security, improved nutrition and affordable healthy diets for all.

[16] Nalty CC, Sharkey JR, Dean WR (2013). School-based nutrition programs are associated with reduced child food insecurity over time among Mexican-origin mother-child dyads in Texas Border Colonias. J Nutr.

[17] U.S. Department of Agriculture: U.S. household food security survey module: three-stage design, with screeners. 2012. [cited 8 August, 2021]. Available at https://www.ers.usda.gov/media/8271/hh2012.pdf.

[18] Smith LP, Hua J, Seto E, Du S, Zang J, Zou S, Popkin BM, Mendez MA (2014). Development and validity of a 3-day smartphone assisted 24-hour recall to assess beverage consumption in a Chinese population: a randomized cross-over study. Asia Pac J Clin Nutr.

[19] Freedman LS, Guenther PM, Krebs-Smith SM, Dodd KW, Midthune D. A population’s distribution of Healthy Eating Index-2005 component scores can be estimated when more than one 24-hour recall is available. J Nutr. 2010;140:1529–34. 10.3945/jn.110.124594 PMC2903306 20573940

[20] Tooze JA, Midthune D, Dodd KW, Freedman LS, Krebs-Smith SM, Subar AF, Guenther PM, Carroll RJ, Kipnis V (2006). A new statistical method for estimating the usual intake of episodically consumed foods with application to their distribution. J Am Diet Assoc.

[21] Zhang S, Midthune D, Guenther PM, Krebs-Smith SM, Kipnis V, Dodd KW, Buckman DW, Tooze JA, Freedman L, Carroll RJ (2011). A NEW MULTIVARIATE MEASUREMENT ERROR MODEL WITH ZERO-INFLATED DIETARY DATA, AND ITS APPLICATION TO DIETARY ASSESSMENT. Ann Appl Stat.

[22] Overview of the Methods & Calculations [https://epi.grants.cancer.gov/hei/hei-methods-and-calculations.html]

[23] Price C, Hamilton W, Cook J, Bickel G MN (2000). Guide to Measuring Household Food Security, Revised 2000.

[24] the poverty guidelines updated periodically in the Federal Register by the U.S. Department of Health and Human Services under the authority of 42 U.S.C. 9902(2). [https://aspe.hhs.gov/topics/poverty-economic-mobility/poverty-guidelines/prior-hhs-poverty-guidelines-federal-register-references/2019-poverty-guidelines]

[25] 2019 Poverty Guidelines [https://aspe.hhs.gov/2019-poverty-guidelines]

[26] Jung NM, de Bairros FS, Pattussi MP, Pauli S, Neutzling MB (2017). Gender differences in the prevalence of household food insecurity: a systematic review and meta-analysis. Public Health Nutr.

[27] Leblanc V, Bégin C, Corneau L, Dodin S, Lemieux S (2015). Gender differences in dietary intakes: what is the contribution of motivational variables?. J Hum Nutr Diet.

[28] Myers AM, Painter MA (2017). Food insecurity in the United States of America: an examination of race/ethnicity and nativity. Food Security.

[29] Popkin BM, Siega-Riz AM, Haines PS (1996). A Comparison of Dietary Trends among Racial and Socioeconomic Groups in the United States. N Engl J Med.

[30] Nord M, Coleman-Jensen A, Gregory C (2014). Prevalence of U.S. Food Insecurity Is Related to Changes in Unemployment, Inflation, and the Price of Food.

[31] Smed S, Tetens I, Bøker Lund T, Holm L, Ljungdalh Nielsen A (2018). The consequences of unemployment on diet composition and purchase behaviour: a longitudinal study from Denmark. Public Health Nutr.

[32] Gundersen C, Ziliak JP (2015). Food Insecurity And Health Outcomes. Health Aff.

[33] French SA, Tangney CC, Crane MM, Wang Y, Appelhans BM (2019). Nutrition quality of food purchases varies by household income: the SHoPPER study. BMC Public Health.

[34] Thompson FE, Larkin FA, Brown MB (1986). Weekend-weekday differences in reported dietary intake: The nationwide food consumption survey, 1977–78. Nutr Res.

[35] Shanks CB, Harden S (2016). A Reach, Effectiveness, Adoption, Implementation, Maintenance Evaluation of Weekend Backpack Food Assistance Programs. Am J Health Promot.

[36] Wilde PE, Ranney CK (2000). The Monthly Food Stamp Cycle: Shopping Frequency and Food Intake Decisions in an Endogenous Switching Regression Framework. Am J Agr Econ.

[37] Shahar DR, Yerushalmi N, Lubin F, Froom P, Shahar A, Kristal-Boneh E (2001). Seasonal Variations in Dietary Intake Affect the Consistency of Dietary Assessment. Eur J Epidemiol.

[38] Poskute AS, Nzesi A, Geliebter A (2021). Changes in food intake during the COVID-19 pandemic in New York City. Appetite.

[39] Magarey A, Mauch C, Mallan K, Perry R, Elovaris R, Meedeniya J, Byrne R, Daniels L (2016). Child dietary and eating behavior outcomes up to 3.5 years after an early feeding intervention: The NOURISH RCT. Obesity.

[40] Willett W. Nutritional epidemiology. Oxford university press; 2013. 10.1093/acprof:oso/9780199754038.001.0001.

[41] U.S. Department of Agriculture FaNS, Center for Nutrition Policy and Promotion: Average Healthy Eating Index-2015 Scores for Americans by Poverty Income Ratio (PIR)*. What We Eat in America, NHANES 2015–2016. 2020.

[42] DeSalvo KB, Olson R, Casavale KO (2016). Dietary Guidelines for Americans. JAMA.

[43] Ansai N, Wambogo EA. Fruit and vegetable consumption among adults in the United States, 2015–2018. NCHS Data Brief, no 397. Hyattsville, MD: National Center for Health Statistics. 2021. 10.15620/cdc:100470externalicon. 33541518

[44] Aggarwal A, Monsivais P, Cook AJ, Drewnowski A (2011). Does diet cost mediate the relation between socioeconomic position and diet quality?. Eur J Clin Nutr.

[45] Goodman M, Thomson J, Landry A (2020). Food Environment in the Lower Mississippi Delta: Food Deserts, Food Swamps and Hot Spots. Int J Environ Res Public Health.

[46] Hager ER, Cockerham A, O’Reilly N, Harrington D, Harding J, Hurley KM, Black MM. Food swamps and food deserts in Baltimore City, MD, USA: associations with dietary behaviours among urban adolescent girls. Public Health Nutr. 2017;20:2598–607. 10.1017/S1368980016002123 PMC5572508 27652511

[47] Begley A, Paynter E, Butcher LM, Dhaliwal SS. Examining the Association between Food Literacy and Food Insecurity. Nutrients. 2019;11(2):445. 10.3390/nu11020445. 10.3390/nu11020445 PMC6412525 30791670

[48] Ranjit N, Macias S, Hoelscher D (2021). Factors related to poor diet quality in food insecure populations. Translational Behavioral Medicine.

[49] Bhattacharya J, DeLeire T, Haider S, Currie J (2003). Heat or eat? Cold-weather shocks and nutrition in poor American families. Am J Public Health.

[50] Drewnowski A (2010). The cost of US foods as related to their nutritive value. Am J Clin Nutr.

[51] Whiting E, Ward C (2010). Food Provisioning Strategies, Food Insecurity, and Stress in an Economically Vulnerable Community: The Northern Cheyenne Case. Agric Hum Values.

[52] Moon S, Kang MY, Bae YH, Bodkin CD (2018). Weather sensitivity analysis on grocery shopping. Int J Mark Res.

[53] Moon S, Kwon J, Jung S-U, Bae YH (2018). The impact of individual differences in weather sensitivity on weather-related purchase intentions. Int J Mark Res.

[54] Chu IY, Alam P, Larson HJ, Lin L. Social consequences of mass quarantine during epidemics: a systematic review with implications for the COVID-19 response. J Travel Med. 2020;27(7):taaa192. 10.1093/jtm/taaa192. 10.1093/jtm/taaa192 PMC7649384 33051660

[55] Weersink A, von Massow M, Bannon N, Ifft J, Maples J, McEwan K, McKendree MGS, Nicholson C, Novakovic A, Rangarajan A (2021). COVID-19 and the agri-food system in the United States and Canada. Agricultural Systems.

[56] Niles MT, Bertmann F, Belarmino EH, Wentworth T, Biehl E, Neff R (2020). The Early Food Insecurity Impacts of COVID-19. Nutrients.

[57] Dietz WH (1995). Does Hunger Cause Obesity?. Pediatrics.

[58] Drewnowski A, Specter SE (2004). Poverty and obesity: the role of energy density and energy costs. Am J Clin Nutr.

[59] Zizza CA, Duffy PA, Gerrior SA (2008). Food insecurity is not associated with lower energy intakes. Obesity (Silver Spring).

[60] Ravelli MN, Schoeller DA. Traditional Self-Reported Dietary Instruments Are Prone to Inaccuracies and New Approaches Are Needed. Front Nutr. 2020;7:90. 10.3389/fnut.2020.00090. 10.3389/fnut.2020.00090 PMC7350526 32719809

