# Supplementary material for: Daily food insecurity is associated with diet quality, but not energy intake, in winter and during COVID-19, among low-income adults
Source: Nutr J. 2022 Mar 24;21:19. doi: 10.1186/s12937-022-00768-y (PMC8943349; doi:10.1186/s12937-022-00768-y)
Supplement: Supplementary file 1 — Additional file 1: Table S1. The adjusted associations between food insecurity score and food/nutrient intake, diet quality, and HEI component scores in females. [file 12937_2022_768_MOESM1_ESM.docx]

**Supplemental Table 1:** The adjusted associations between food insecurity score and food/nutrient intake, diet quality, and HEI component scores in females^1^

|  | **All^2^** | | **Fall^3^** | | **Winter^3^** | | **Before Covid-19^4^** | | **During Covid-19^4^** | |
| --- | --- | --- | --- | --- | --- | --- | --- | --- | --- | --- |
|  | Coefficient  (95% CI) | P-value | Coefficient  (95% CI) | P-value | Coefficient  (95% CI) | P-value | Coefficient  (95% CI) | P-value | Coefficient  (95% CI) | P-value |
| N of person-days | 274 |  | 154 |  | 120 |  | 171 |  | 103 |  |
| **Total diet** |  |  |  |  |  |  |  |  |  |  |
| Total energy, kcal^5^ | 1.25  (-112.00, 114.20) | 0.98 | -37.22  (-160.00, 85.50) | 0.55 | -1.50  (-192.00, 188.70) | 0.99 | 40.60  (-92.00, 173.30) | 0.54 | -116.30  (-271.00, 38.30) | 0.14 |
| HEI, score^5^ | -2.37  (-4.82, 0.08) | 0.06 | -1.01  (-3.86, 1.84) | 0.49 | **-6.18**  **(-10.26, -2.10)** | **0.003** | -1.93  (-6.13, 2.28) | 0.37 | **-3.87**  **(-6.19, -1.55)** | **0.001** |
| **Food and food group** |  |  |  |  |  |  |  |  |  |  |
| Total fruit, cup | **-0.11**  **(-0.20, -0.02)** | **0.02** | **-0.11**  **(-0.21, -0.01)** | **0.04** | -0.12  (-0.24, 0.01) | 0.06 | **-0.22**  **(-0.39, -0.05)** | **0.008** | 0.08  (-0.07, 0.22) | 0.29 |
| Whole fruit, cup | **-0.09**  **(-0.17, -0.01)** | **0.04** | -0.08  (-0.17, 0.01) | 0.06 | **-0.15**  **(-0.26, -0.03)** | **0.01** | **-0.17**  **(-0.34, -0.01)** | **0.05** | 0.09  (-0.08, 0.25) | 0.30 |
| Total vegetable, cup | 0.05  (-0.22, 0.32) | 0.72 | 0.08  (-0.25, 0.41) | 0.62 | -0.07  (-0.35, 0.21) | 0.65 | -0.14  (-0.42, 0.15) | 0.36 | 0.25  (-0.01, 0.49) | 0.06 |
| Greens and beans, cup | -0.05  (-0.11, 0.01) | 0.08 | -0.05  (-0.10, 0.01) | 0.12 | -0.07  (-0.16, 0.02) | 0.14 | **-0.08**  **(-0.13, -0.02)** | **0.004** | -0.07  (-0.14, 0.01) | 0.09 |
| Whole grains, oz | **-0.28**  **(-0.52, -0.05)** | **0.02** | -0.15  (-0.37, 0.08) | 0.20 | **-0.65**  **(-1.09, -0.22)** | **0.003** | -0.23  (-0.58, 0.13) | 0.21 | **-0.48**  **(-0.88, -0.09)** | **0.02** |
| Dairy, cup | **0.23**  **(0.08, 0.39)** | **0.003** | **0.26**  **(0.13, 0.39)** | **<0.001** | 0.28  (-0.13, 0.69) | 0.19 | 0.16  (-0.02, 0.33) | 0.07 | **0.24**  **(0.08, 0.41)** | **0.003** |
| Total protein, oz | -0.27  (-0.85, 0.32) | 0.37 | -0.45  (-0.90, 0.01) | 0.06 | 0.06  (-0.86, 0.98) | 0.90 | -0.14  (-0.81, 0.53) | 0.69 | -0.42  (-1.02, 0.18) | 0.17 |
| Seafood/plant protein, oz | 0.02  (-0.4, 0.5) | 0.94 | 0.08  (-0.39, 0.55) | 0.74 | 0.06  (-0.36, 0.49) | 0.77 | 0.27  (-0.39, 0.92) | 0.42 | -0.41  (-0.95, 0.13) | 0.13 |
| Refined grains, oz | **0.53**  **(0.20, 0.85)** | **0.002** | 0.29  (-0.08, 0.66) | 0.13 | **0.83**  **(0.22, 1.44)** | **0.007** | **0.53**  **(0.15, 0.92)** | **0.005** | 0.54  (-0.08, 1.15) | 0.09 |
| Salt, g | **0.17**  **(0.01, 0.33)** | **0.04** | 0.12  (-0.06, 0.30) | 0.21 | 0.20  (-0.04, 0.44) | 0.10 | -0.01  (-0.17, 0.17) | 0.99 | **0.31**  **(0.14, 0.47)** | **<0.001** |
| Added sugars, % of energy | -9.65  (-23.19, 3.90) | 0.16 | 7.31  (-12.55, 27.18) | 0.47 | -30.66  (-63.34, 2.02) | 0.07 | -2.20  (-32.26, 27.86) | 0.89 | -2.20  (-32.26, 27.86) | 0.89 |

1. CI: confidence interval; HEI: healthy eating index
2. Adjusted for gender, race, employment, poverty status, COVID-19 months, seasons, total energy intake, study weeks, and weekdays
3. Adjusted for gender, race, employment, poverty status, COVID-19 months, total energy intake, study weeks, and weekdays
4. Adjusted for gender, race, employment, poverty status, seasons, total energy intake, study weeks, and weekdays
5. GEE models examining the association of daily FI scores and total energy intake and HEI score were not adjusted for total energy intake
